# Comparison of Laboratory Methods for the Clinical Follow Up of Checkpoint Blockade Therapies in Leukemia: Current Status and Challenges Ahead

**DOI:** 10.3389/fonc.2022.789728

**Published:** 2022-01-27

**Authors:** Basak Aru, Mojdeh Soltani, Cemil Pehlivanoglu, Ege Gürlü, Mazdak Ganjalikhani-Hakemi, Gülderen Yanikkaya Demirel

**Affiliations:** ^1^Department of Immunology, Faculty of Medicine, Yeditepe University, Istanbul, Turkey; ^2^Department of Immunology, Faculty of Medicine, Isfahan University of Medical Sciences, Isfahan, Iran; ^3^Department of Emergency Medicine, Hatay Training and Research Hospital, Antakya, Turkey; ^4^Faculty of Medicine 4^th^Year Student, Yeditepe University, Istanbul, Turkey

**Keywords:** immune checkpoint blockade, immune checkpoint inhibitors (CPI), leukemia, clinical laboratory (cl), immune check point

## Abstract

The development of immune checkpoint inhibitors, the monoclonal antibodies that modulate the interaction between immune checkpoint molecules or their ligands on the immune cells or tumor tissue has revolutionized cancer treatment. While there are various studies proving their efficacy in hematological malignancies, there is also a body of accumulating evidence indicating that immune checkpoint inhibitors’ clinical benefits are limited in such diseases. In addition, due to their regulatory nature that balances the immune responses, blockade of immune checkpoints may lead to toxic side effects and autoimmune responses, and even primary or acquired resistance mechanisms may restrict their success. Thus, the need for laboratory biomarkers to identify and monitor patient populations who are more likely respond to this type of therapy and the management of side effects seem critical. However, guidelines regarding the use of immune checkpoint inhibitors in hematological cancers and during follow-up are limited while there is no consensus on the laboratory parameters to be investigated for safety and efficacy of the treatment. This review aims to provide an insight into recent information on predictive and prognostic value of biomarkers and laboratory tests for the clinical follow up of hematological malignancies, with an emphasis on leukemia.

## 1 Introduction

Therapeutic approaches targeting immunosuppressive features of the tumor microenvironment (TME) or cancer cells may reinforce antitumor immune responses in both solid and hematological malignancies. Up-regulation of inhibitory checkpoints on the surface of innate and acquired immune cells is considered as one of the important immunosuppressive features in most types of cancers (1). After the approval of cytotoxic T lymphocyte-associated molecule-4 (CTLA-4) inhibitor ipilimumab in 2011, immune checkpoint inhibitors (ICIs) have revolutionized cancer treatment (2). Under physiological conditions, immune checkpoint proteins (ICPs) that involve in various inhibitory and stimulatory pathways regulate the homeostasis between pro-inflammatory and anti-inflammatory signals while in cancer, the malignant cells promote an immunosuppressive tumor microenvironment to diminish anti-tumor immune response for immune evasion. Currently, clinically approved ICIs target either CTLA-4 or PD-1 signaling pathways while others targeting TIM3 and LAG-3 are currently under investigation (3, 4). CTLA-4 and PD-1 pathways differ from each other according to the periods of the immune response they involve (5). CTLA-4 expression is restricted to T cells, inhibiting cellular activation at the initial stages, mainly in lymph nodes while PD-1, expressed on T cells, B cells and myeloid cells regulates activated T cells in peripheral tissues. In summary, activation of both ICPs exerts similar effects on T-cell activity but their signaling mechanisms as well as the location where cellular interactions take place differ.

Immune checkpoint blockade (ICB) refers to usage of monoclonal antibodies which target immune checkpoint molecules or their ligands on the surface of immune and/or tumor cells which act by hampering the inhibitory mechanisms on T cells in addition to activating cells taking part in innate and adaptive immunity to exert an effective anti-tumor response (6, 7). This immunotherapeutic approach was first approved by FDA for the treatment of melanoma and non-small cell lung cancer (NSCLC), and its beneficial effects in treatment of hematological malignancies have also been proven (8–10). Historically, the promising effects of immunotherapy in the field of hematological malignancies have been initially recognized after success of stem cell transplantation (SCT) as well as various mAbs against tumor surface proteins. Accordingly, accumulating data have demonstrated that due to some genetic factors, the physiologic functions of inhibitory checkpoints are dysregulated in several hematologic diseases such as myelodysplastic syndrome (MDS), lymphoid neoplasms and acute myeloid leukemia (AML) (11). ICB therapies in hematological malignancies have primarily started with anti-CTLA-4 (Ipilimumab) and anti PD-1/PD-L1 antibodies (Pembrolizumab, Nivolumab, Atezolizumab, Avelumab, and Durvalumab) and there are numerous clinical trials evaluating their therapeutic benefits alone or in combination with other treatments (**Table S1**); however, they have shown clinical benefits mostly in certain types of hematological malignancies. Moreover, blockade of other inhibitory checkpoints such as LAG-3, TIM-3, and TIGIT or some innate checkpoints on NK cells and macrophage with therapeutic agents are under clinical evaluation as a potential approach to overcome acquired resistance (11–13). Some ongoing studies are investigating efficacy of ICB in association with other immunotherapies such as CAR-T cell therapy, with promising outcomes (14, 15).

Despite favorable clinical benefits of these immunotherapies in most types of hematologic malignancies, ICB is used only in the treatment of certain tumor types which have high infiltration of immune cells. Furthermore, due to some primary or acquired resistance mechanisms, checkpoint blockade therapies (CBTs) on their own are not able to achieve long-term disease control in leukemia, and thus, certain patients diagnosed with leukemia may not respond well to this treatment or benefit from it only during the initial stage. On the other hand, as inhibitory checkpoints play role in maintaining the balance of immune function, patients treated with these agents may suffer from a series of side effects called immune-related adverse events (irAEs) (16). Due to high cost of CBT, its use only in certain leukemia patients, and its potential toxicities or adverse effects, there is an unmet need for assessment of laboratory biomarkers to identify and monitor patient populations who are more likely respond to this type of therapy and management of side effects. Despite availability of several potential biomarkers and laboratory approaches, further studies are required since still there is no consensus. In solid tumors, checkpoints or their ligands’ expression levels in the tumor tissue as well as the serum levels of their soluble forms are usually evaluated, however in case of hematologic malignancies, it is more complex and identification of reliable predictive biomarkers is more difficult (17). Here, we aimed to review recent information on the predictive and prognostic value of laboratory methods and biomarkers for clinical follow-up of patients with leukemia or lymphoma who were treated with ICBs, particularly with anti-CTLA-4 and anti-PD-1/PD-L1 antibodies, the most widely used ICIs.

## 2 General Outlook of Adverse Effects of Immune Checkpoint Blockade Therapies

As mentioned above, regarding the physiologic role of ICIs in maintaining the immune system in balance, their blockade might result in several side effects and toxicities and/or provoke autoimmune reactions in both solid and hematologic malignancies. They include a wide spectrum of adverse effects including dermatologic, gastrointestinal, and hepatic disorders, iritis, hypophysitis, pneumonitis and endocrine events. These treatment-related complications which are usually less frequent in PD-1/PDL1 blocking compared to CTLA-4, are managed based on their grade. Even though it has been reported that some endocrine complications have resulted in permanent organ dysfunction necessitating hormone replacement therapy, other irAEs have typically been reversible and manageable (18, 19). Here, we describe some more common immune-related adverse events that may occur following CBT in hematological malignancies.

Previous studies have shown that blockade of PD-1/PD-L1 pathway is usually well-tolerated and associated with less adverse effects. Several studies have investigated adverse events with anti-PD-1 therapies during treatment of hematologic malignancies and have shown that most patients (approximately 78%) experience minor adverse events in the form of rash and thrombocytopenia, followed by fatigue, diarrhea, nausea, pruritus, and pyrexia. Approximately 22% of patients experienced Grade 3 or higher adverse events such as lymphopenia, stomatitis, increased lipase, MDS and pancreatitis. Such irAEs were reported in the treatment of diffuse large B-cell lymphoma (DLBCL) and Hodgkin lymphoma (HL) with *pembrolizumab*, where the most common side effect was neutropenia, followed by fatigue, diarrhea, and upper respiratory infections. Other studies indicated that anti-PD-1 therapy is associated with pancreatitis, lymph node pain, pneumonitis, and MDS (20–24). Furthermore, some of these studies have demonstrated Grade 3-4 neutropenia and thrombocytopenia (19% and 8%, respectively) following administration of pidilizumab (an anti-PD-1 antibody) after ASCT in patients with DLBCL, primary mediastinal B-cell lymphoma (PMBCL), and transformed indolent NHL, which are attributable to autoimmune etiology, but further studies are required to confirm this finding (20).

During treatment of hematological malignancies with anti-CTLA-4 antibodies (most commonly Ipilimumab), the most common Grade 3 adverse events were diarrhea and fatigue. Furthermore, AST elevation and thrombocytopenia have been also reported less frequently. Additionally, some other trials have evaluated efficacy and safety of ipilimumab in patients who had failed allo-SCT. Interestingly, administration of ipilimumab in hematopoietic malignancy after HSCT demonstrated a similar profile of toxicities (22). Davids et al. have investigated adverse events after use of ipilimumab in relapsed hematologic malignancies (including AML, lymphomas, myeloma, myelodysplastic syndrome, and myeloproliferative syndromes) following HSCT. In this study, patients received deferent doses of induction therapy with Ipilimumab (3 or 10 mg per kilogram of body weight every 3 weeks for a total of 4 doses). Of 28 evaluable patients, one case of acute GVHD of the gut and 3 cases of chronic GVHD of the liver were reported (25).

## 3 Role of Clinical Immunology and Hematology Laboratories in Diagnosis and Follow-up of ICI Therapies

Leukemia develops in bone marrow and its surrounding microenvironment, which is a heterogenous environment with stromal cells, blood vessels, immune cells, and extracellular matrix (ECM) (26). It is easier to obtain a sample compared to solid tumors, however its heterogeneity, the need for expertise and the cost makes it challenging for clinical laboratories. Advanced technical expertise required for immunological assays that is centralized to advanced laboratories can also be considered as a drawback for hospital-based clinical laboratories.

There are three main mechanisms of resistance developed against ICI therapies; primary, adaptative and secondary or acquired resistance (26). Based on this classification, “primary” mechanism is referred to pre-existing resistance to immunotherapy and usually contains patients who do not respond at all to immunotherapeutic treatments, however, appearance of resistance as a Darwinian mechanism of adaptation is considered as “adaptive”. Finally, “acquired” (also called “secondary”) means appearance of resistance after immunotherapy instigation after a transient period of disease control. To understand and predict these mechanisms, there is a need for defining the “immune contexture” by analyzing the spatial localization, density, and functional orientation of immune cell populations in bone marrow and surrounding tissues (26). Routine immunophenotyping for leukemias and other hematological malignancies is an essential part of the diagnostic work-up. There are also some biomarkers that have been suggested for use in different types of resistance mechanisms such as myeloid-derived suppressor cells (MDSCs), tumor associated macrophages (TAMs) and tumor infiltrating cells (TILs) for solid tumors (26), yet there is not enough evidence to use these markers in clinical follow-up of leukemias.

**Figure 1:**
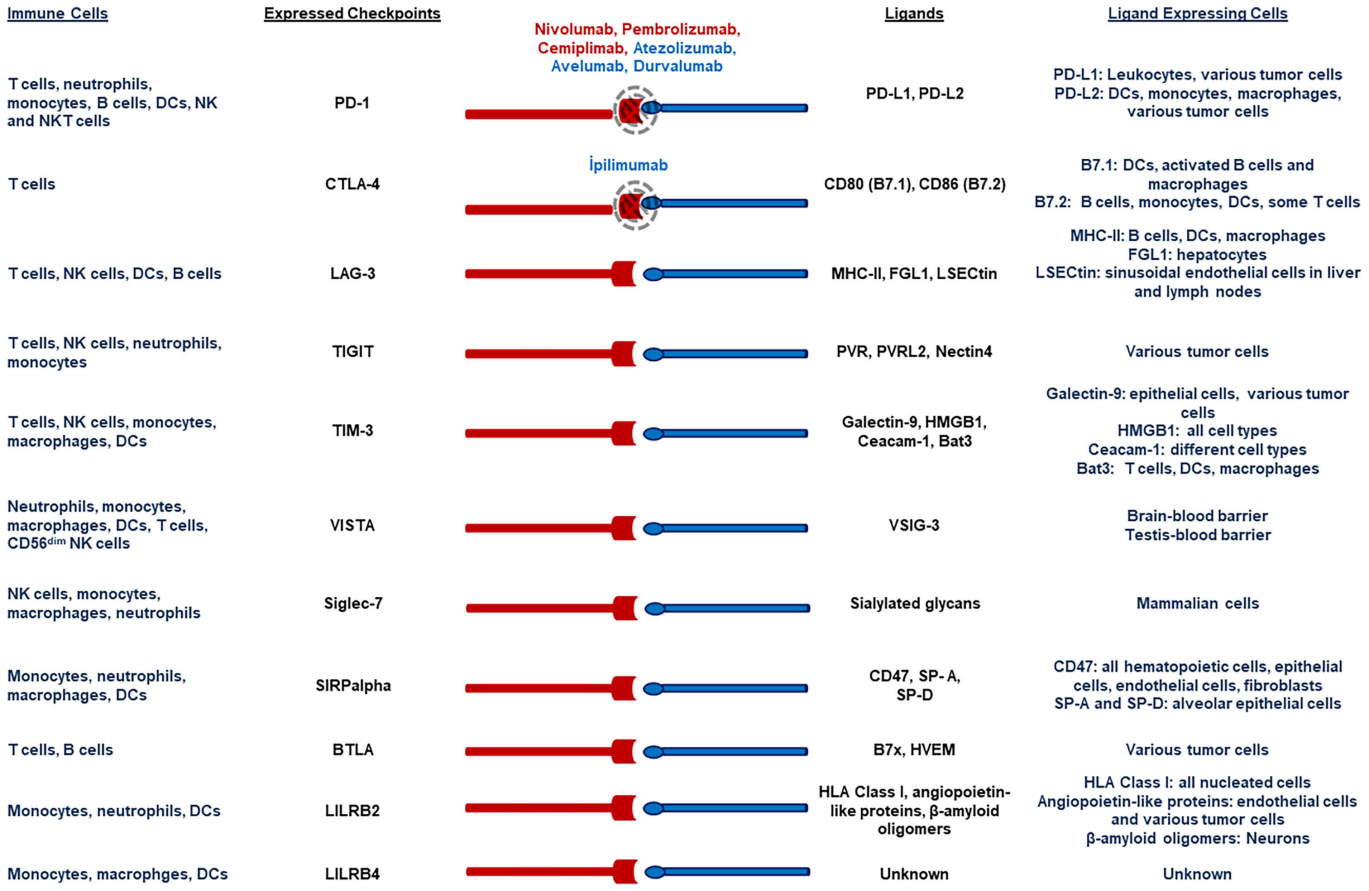
Checkpoint markers on the immune cells and their counterparts on the cells.

The type of cells expressing immune checkpoint markers and their ligands are given in **Figure 1**. As will be observed, only drugs blocking PD-1/PD-L1, and CTLA-4/CD80-CD86 have been currently approved for clinical use. Other ICIs are on the line, and we need to explore the roles of these markers for clinical laboratories as well.

## 4 Clinical Tests for Selection and Evaluation of Leukemia Patients in Checkpoint Blockade Therapies

This review is focused on immunology- and hematology-related tests for ICIs since the biochemistry tests which are an important part of the follow-up are handled by other laboratory specialists, and they require extensive knowledge in these tests (**Table 1**).

**Table 1:** Suggested immunological tests for follow-up of patients administrating immune checkpoint inhibitors.

Name of Test	Brief Explanation
Neutrophil-to-Lymphocyte Ratio (NLR)	Neutrophil-to-Lymphocyte Ratio is obtained by dividing neutrophil counts to lymphocyte counts measured in peripheral blood. NLR indicates the balance between acute and chronic inflammation. Patients who are on immune checkpoint inhibitors should be tested for neutrophil-to-lymphocyte ratio regularly.
Immunophenotyping by cytometry	Immunophenotyping detects the presence and expression of target cell protein. To detect the expression levels of immune checkpoints, evaluations at protein levels will be useful for follow-up.
Immunohistochemistry assays (IHC)	Most of the pathological evaluation is based on IHC tests. Few are used in immunology/hematology laboratories.
Enzyme Linked ImmunoSorbent Assays (ELISAs)	ELISA is an immunoassay performed by using an enzyme labeled immunoreactant and immunosorbent. ELISA tests can be used to detect antibodies developed against immune checkpoint inhibitors.
Receptor-receptor ligand assay	Receptor ligand assay is used to quantify receptors and ligands, and the method that is used can be either radioactive or non-radioactive. Cytometry is an easier method, if available.
Stimulation tests	Lymphocyte stimulation test is used to determine the ability of the lymphocytes to respond to a stimulus *in vitro*.
T-cell proliferation assays	The aim of cell proliferation assay is evaluating whether cells are induced to proliferate after giving a specific stimulus. In addition to that, T cell proliferation assay can be used to determine the potential of proliferation of different cell populations in response to the same stimulus.
Cytokine production assays	Cytokine production assay identifies live cytokine secreting cells. Serum cytokine levels can be detected by bead based assays with cytometry.
T-cell cytotoxicity assay	Especially antibody dependent cell cytotoxicity will provide information on status of the immune system.
Anti-drug antibodies	Anti-drug antibodies that are formed against the immune checkpoint inhibitor drugs can be detected by commercial ELISA tests. Anti-drug antibodies can decrease the efficacy and amount of the drug that is available.
Electrochemiluminescence (ECL)	Electrochemiluminescence (ECL) is used to detect to antigen or antibody by the help of the change in ECL signal. ECL can be used as an alternative for ELISA to detect anti-drug antibodies (ADA).

### 4.1 Determination of Leukemia Subtype and Checkpoint Markers at Diagnosis and Follow-Up

Leukemia subtypes are determined by using a combination of morphology, pathological findings based on morphology and immunohistochemistry, immunophenotyping, cytogenetics and molecular testing. All these tests require a high level of expertise throughout all stages of analysis, evaluation, and reporting. As we follow the response and relapse by morphology, immunohistochemistry, next generation cytometry and molecular testing in leukemias, adding checkpoint biomarkers to these routine assessments may contribute to ICIs therapy. There are FDA-approved ICIs tests such as PD-L1/PD-1 immunohistochemistry testing and Microsatellite Instability (27), and they all have specific limitations for leukemias that will be discussed in detail throughout this article.

PD-L1 expression on cells is considered a selection criterion for some solid tumors, but we do not yet have enough evidence on different subtypes of leukemia and their PD-L1 expression levels. Some researchers suggest that PD-L1 is a still imperfect biomarker since responses are observed in patients with low or negative expression tumors. While PD-L1 expression can be a predictive marker for one tumor subtype of the same tissue, it can also be a prognostic biomarker in leukemias as in lung cancers (28, 29). Currently, our knowledge regarding the involvement of PD-L1 in hematological malignancies are less investigated in comparison with solid tumors, and mostly related to the classical Hodgkin’s lymphoma (cHL), which has different characteristic features compared to AML (30–32). In terms of leukemia, Brodská et al. have reported the relationship between high PD-L1 expression and worse outcome in the presence of NPM1 and FLT3 mutations (30). Dong et al. indicated PD-L1 presence on AML cells induce T regulatory cells, whose frequency are known to increase in AML to exert their suppressive functions *via* promoting Foxp3 and PD-1 expression (33, 34). Rezayeen et al. underlined the role of PD-1/PD-L1 axis in drug resistance, suggesting its’ blockade may also promote chemotherapy sensitivity (35). Jimbu et al. mentioned the blockade of PD-1/PD-L1 axis in combination with hypomethylation agents have shown promising results while their administration as monotherapies failed, both in *de novo* and relapsed AML (32).

In contrast to PD-L1 expression which can be considered as a marker for certain solid tumors, the potential benefits of CTLA-4 targeted therapies’ cannot be predicted due to the (5) proteins’ low basal expression levels as well as the common presence of its’ ligand B7 (5). However, CTLA-4 involvement in chronic lymphocytic leukemia (CLL) (36–38) and ALL (39) have previously been reported. In AML, certain polymorphisms of the CTLA-4 gene have been associated with leukemic relapse as well as decreased survival (40).

### 4.2 Immunohistochemistry Tests for Detection of ICI Responder Patients

Even for a simple antibody staining for PD-L1, there are technical difficulties such as clone of the antibodies, staining method, threshold levels for positive/negative, and standardization of assays. Choosing the most appropriate clone is a serious task for clinical laboratory staff, since a comparative study (41) on lung cancer samples implies that different clones have different performances and for different products, their specific clones of PD-L1 is required, which makes the process complicated. These differences in performance have been shown with epitope mapping experiments, and they may be attributed to different isoforms of PD-L1 (41). Further difficulties are expected in bone marrow and peripheral blood samples of leukemia patients due to heterogeneity of cell populations in samples. The FDA approved PD-L1 test is performed with a device (PD-L1 IHC 22C3 PharmDx assay) that uses two parameters; Tumor Proportion Score (TPS), which is the percentage of PD-L1 positive tumor cells in relation to viable tumor cells within the sample, and Combined Proportion Score (CPS), which identifies all PD-L1 positive cells in the sample including tumor cells, lymphocytes, and macrophages by membrane staining. There are cut-off values determined for solid tumors for these biomarkers such as TPS ≥1% and CPS ≥10% (27). At the moment, five different assays have FDA approval for these tests (42). Since most of available information is obtained through solid tissue research, clinical immunology and hematology laboratories may only implement these testing during their routine leukemia diagnostics after obtaining evidence from further research.

### 4.3 Tumor Mutational Burden

Higher tumor mutational burden is associated with a higher level of immunogenic neopeptides that would stimulate T cells in tumor microenvironment (43). A TMB value of ≥10 mutations/Mb is used as a predictive factor. Although FDA has approved this test as a companion diagnostic test (27), there are controversial results from different studies and manuscripts on predictive failure of this test in ICI treatments (44).

### 4.4 Microsatellite Instability

Microsatellite instability (MSI) is considered a marker for immunotherapy of solid tumors, but there are controversial views on the occurrence of MSI in hematological malignancies. In a study performed on 39 different cancer types, microsatellite instability was low in patients with chronic lymphocytic leukemia (CLL) (MSI-H=0.30), while patients with acute myeloid leukemia (AML) had zero MSI values (45).

### 4.5 Immunophenotyping of Tumor Cells and T-Cells in Bone Marrow

Innate immune response and antigen presentation are crucial for anti-leukemic immune response, while effective protection may not be obtained and sustained without effector T cells (46). In this respect, detailed immunophenotyping of effector T cells in bone marrow samples (aspirates and biopsies) along with leukemia immunophenotyping during follow up will contribute to our understanding of immune response in immunotherapies. A flow cytometric panel, consisting of two tubes suitable for 13-color flow cytometry instruments for the evaluation of ICPs in leukemia samples is presented on **Table 2** (47). In this panel, lymphocytes can be identified according to their forward scatter/side scatter localization, followed by the determination of common leukocyte antigen positivity by CD45 vs. side scatter gating. T cells and B cells can be differentiated by CD19 vs. CD3 plot and T cells can further be classified according to CD4+ (T helper) and CD8+ (T cytotoxic) expressions. Effector memory T cells can be differentiated between central memory cells (CD3+CCR7+) and effector memory cells (CD3+CCR7-) according to their CCR7 expression (48). CD28 positivity on CD3+CD8+ T cells identifies IFN-γ–producing, highly cytotoxic T cells (49) while CD95 on T cell gate identifies apoptotic cell population (50). Monocytes and NK cells can be discriminated according to CD14 vs. CD16 and CD14 vs. CD56 expressions, respectively. In the second tube, activation of CD3+CD4+ or CD3+CD8+ cells, identified from the lymphocyte gate can be analyzed by CD39 and CD69 expressions in addition to determination of tumor reactivity by the expression of CD137 (51–53). Similarly, expression levels of ICPs PD-1, PD-L1, CTLA-4, LAG3 and TIM3 on lymphocytes can be evaluated from the lymphocyte gate. The combination of CD45RO and CD45RA expression on lymphocyte gate can be used to identify naive T (CD45RA+) and primed/memory cells T (CD45RO) cells on either CD3+CD4 or CD3+CD8+ gates (54). In addition to use of the panel mentioned, a bone marrow aspirate is necessary to have a baseline status of ICPs at diagnosis and each time in order to measure outcome of the ICI therapies. Suggested parameters for this approach is presented in **Table 3**. Yet, due to the complex nature of flow cytometric analyses, standardized procedures are required for reproducibility (61).

**Table 2:** Suggested panel for the evaluation of immune checkpoints in leukemia samples.

Ex*	488 nm					638 nm			405 nm				
FL**	525/40	585/42	610/20	690/50	780/60	660/20	712/25	780/60	450/45	252/40	610/20	660/20	780/60
Tube- 1	CD45-RO	CD95	CD28	7-AAD	CCR7	CD14	CD4	CD3	CD8	CD45	CD19	CD16	CD56
Tube- 2	CD45-RA	PD-1	CD137	CD39	CTLA-4	CD69	CD4	CD3	CD8	CD45	LAG3	TIM3	PD-L1

*Ex, Excitation wavelength; **FL, Fluorescence channel.

**Table 3:** Panel for detection of immune checkpoint proteins.

Antibodies*	Description**	Other Names**	Properties
CD45	Protein tyrosine phosphatase; C receptor type	PTPRC; B220; GP180; LCA; LY5; T200	Pan leukocyte marker, some blasts may have dim staining of CD45
CD3	CD3	CD3D, CD3E, CD3G	Pan T-cell marker
CD28	CD28 antigen	Tp44	Activation marker
CD86	CD28 antigen ligand 2, B7-2 antigen	B7-2; B70; CD28LG2; LAB72; MGC 34413	Also, a ligand for CTLA Leukemia monocytic/dendritic lineage marker (55)
CD134	Tumor necrosis factor receptor superfamily; member 4	OX-40; TNFRSF4; ACT35; TXGP1L	Marker for activated T cells, also expressed by neutrophils. Promotes T cell response and proliferation. *In silico* studies found that it is related to poor prognosis (56)
CD137	Tumor necrosis factor receptor superfamily; member 9	4-IBB; TNFRSF9; CDw137; ILA; MGC2172	Expressed by monocytes and macrophages, enhances their tumoricidal activity (57)
CD152	CTLA-4; cytotoxic T-lymphocyte associated protein 4	CTLA-4	Expressed by activated CD4+ and CD8+ cells, competes with CD80 and CD86. More human data is necessary (58)
CD274	programmed cell death 1 ligand 1	PD-L1; B7-H; B7H1, PDCD1L1	Predicts survival in AML patients (59, 60)
CD279	programmed cell death 1	PD-1; PD-1, SLEB2; hPD-1	Expressed by activated T cells, dendritic cells, and monocytes. Inhibits T cell activation, leads to apoptosis, exhaustion and anergy of T cells. Not found on leukemia cells (59)
HLA-DR		MHC II/Activation marker	

For valid results, each laboratory needs a validation/verification for the panel of the antibodies, analysis protocol, evaluation, and reporting (55–60). *CD Name from Cluster of Differentiation List of HDCM (hdcm.org); **NCBI, National Center for Biotechnology Information (ncbi.nlm.nih.gov) listed description and names.

### 4.6 Markers of Inflammation

Inflammation within the bone marrow is a risk for leukemias and is correlated with disease progression and prognosis (62, 63). Blood levels of neutrophils, lymphocytes, platelet counts and circulating proteins such as C-reactive Protein (CRP) and Interleukin-6 (IL-6) are used as markers of inflammation (64). HLA-DR expression by monocytes has been suggested as a predictive marker for inflammation especially for intensive care unit patients, but has not yet found its place in most of the clinical laboratories (65). Importance of identifying CD14^+^HLA-DR^low/neg^ monocytes in bone marrow of leukemia patients treated with ICIs should be recognized. This type of monocytes has been shown to negatively affect the responses to anti-CTLA-4 and anti-PD-1 treatments (66). Exploration of these differences for different type of leukemias, would add information to our understanding of immune response for ICIs in leukemias.

### 4.7 Humoral Markers

Humoral markers may be used as a surrogate marker of immunity in cancers as they have been used historically. CRP is an acute phase biomarker of inflammation and has been found to be high in diagnostic testing before ICIs therapies (67). It has been suggested that along with conventional biomarkers such as CRP and white blood cell counts; combinations of novel inflammatory parameters to specify organ damage should be developed and used (68). Studies have shown that serial cytokine measurements with serum cytokine signatures may be used as a predictive factor for ICIs therapies, but even though exposure to interferons is critical for PD-1/PD-L1 targeting cancer treatment, pro-inflammatory cytokines such as IL-2 or IFN-γ showed no significance in this respect (29, 67, 69). IFN-γ was reported to be produced by tumor infiltrating lymphocytes (TILs) in solid tumors and shown to upregulate PD-L1 expression on tumor cells (70). Measurement of soluble PD-L1 in patients with lung cancers has been associated with prognosis, and it requires further studies for use of immunotherapies in leukemia.

### 4.8 Host Environment

#### 4.8.1 Microbiome

There are many publications about the effect of microbiome on the development of malignancies, infections, inflammatory and autoimmune diseases. A permissive microbiota is associated with development of hematologic malignancies (acute leukemia, myeloma and lymphoma) and also with other non-malignant hematological diseases (71). Therefore, manipulation of intestinal microbiota is expected to enhance the effectiveness of immune ICIs and reduce adverse effects of therapy (72). While there are many papers supporting this finding for different types of tumors, the number of studies on leukemia-microbiome interaction is limited. According to these studies, dysbiosis of microbiota may be induced by infections, chronic inflammations, epithelial barrier alterations, antigenic changes, and molecular mimicry mechanisms (71).

#### 4.8.2 Anti-Drug Antibodies

Development of anti-drug antibodies (ADAs) after using immunotherapies is an important concern both for clinicians and clinical laboratories, as they are considered as a part of resistance to therapy. ADAs may have an inactivating effect on the ICIs, and lead to diminished targeting, and an increase in the elimination of ADA-drug complexes and an immune response that will increase the risk for toxicity (73). Even though there are controversial studies on the subject, further studies on development and detection of these antibodies will provide more insight to patient-tailored therapies (74, 75).

Different types of ELISA methods, electrochemiluminescence and cytotoxicity tests can be used for detection of ADAs. Each laboratory will need to validate the procedures since none of these tests has not been approved for clinical use yet (74). More detailed information from clinical trials about ADAs and their effect on outcome of leukemia therapy will be helpful to consider ADA testing a routine application for clinical laboratories.

#### 4.8.3 Germline Mutations

**Figure 2:**
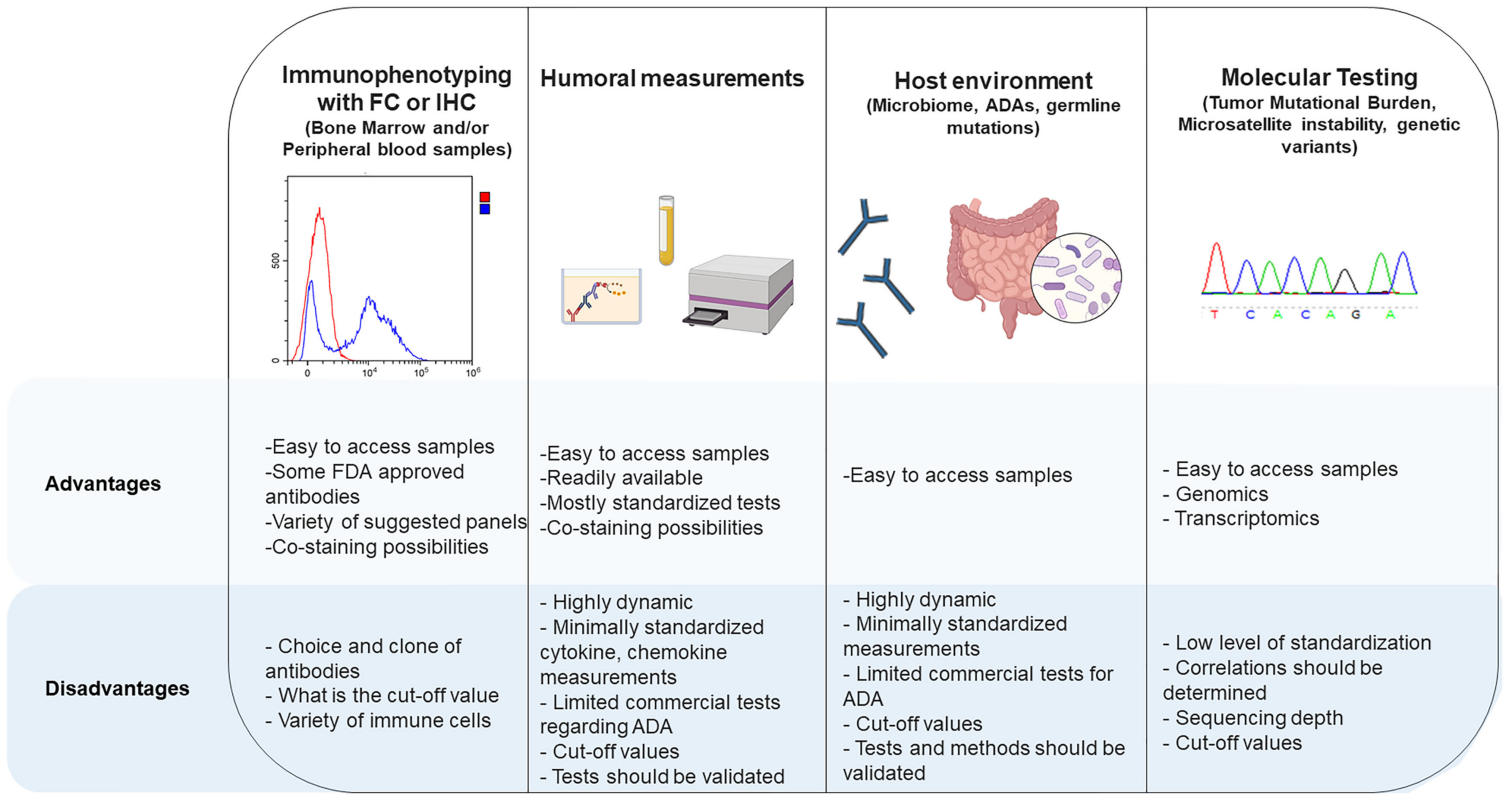
Advantages and disadvantages of different methods for measurement of checkpoint inhibitors and their interactions with other cells.

It has been shown that germline variants are predictive of tumor mutational burden and may be used as a predictive marker for ICIs (76). Inherited pathogenic germline variants in genes coding for CTLA-4 have been shown to react differently to ICIs compared to wild-type CTLA-4 (77). Germline variants in different immune cells also have the potential of modulating ICI response. More investigations on how germline variants influence the immune response to ICI treatments in different types of leukemia will contribute to our understanding of ICI therapies in hematological malignancies. All the tests listed above have their advantages and disadvantages for use in clinical laboratories, which are presented in **Figure 2**.

## 5 Challenges Ahead

A comprehensive study published after analysis of >10,000 cancer samples proposed that there are six different immune subtypes across different cancer types, and wound healing, IFN-γ dominant, inflammatory, lymphocyte depleted, immunologically quiet and TGF-beta dominant subtypes differ by somatic aberrations, microenvironment and survival (78). In another study of two hundred (200) acute myeloid leukemia samples, the results for genetic and epigenetic properties of acute myeloid leukemias were published by The Cancer Genome Atlas Research Network in 2013 (79). Studies designed for understanding the immune landscape of hematological malignancies could contribute to use of ICIs considering these immune signatures. It is noteworthy to emphasize that acute myeloid leukemias have less somatic mutations than other cancer types (18 mutations vs. >50 mutations in other cancer types) (79).

Another challenge for clinical laboratories is bridging the genomic information with other clinical features of leukemias. Germline variants are not always detectable for all leukemias, and there are ongoing studies aiming to gain more precise information on the subject (80, 81). Clinical evaluation and use of these tests are challenging because of the lack of familiarity with this type of testing, the requirement for DNA samples that reasonably approximate the germline state and absence of standardization across the different brands of diagnostic platforms for genes that are sequenced as well as their capabilities for detection of full range of variants (82).

There is an unmet need for development of predictive markers for serious side effects such as myocarditis, encephalitis or acute hypophysitis, which could lead to death. Since the action mechanism of ICI treatments relies on the inhibition of immune response, treatment may end up with inflammation of tissues and organs (83). To be able to make predictions currently, available tests and novel tests for prediction of the inflammation should be included in routine testing. A guideline was developed for response criteria for use in clinical trials testing immunotherapies for solid tumors: iRECIST by Response Evaluation Criteria in Solid Tumors Working Group (RECIST) (84). Up to date, there is limited guideline information for use of ICIs in leukemias and for follow-up therapy in laboratories (85).

An experimental study has reported that the Teffs/Tregs ratio can be used as a predictive marker, and with a higher ratio, a better response is expected (86). Other studies on solid tumors also provide support to this finding (87). Exhausted T cells (CD8^+^PD-1^+^CTLA^+^ cells) are known to influence immune response to immunotherapy, and due to possibility of performing functional measurements such as cytokine measurements, it is possible to identify these exhausted T cells in leukemias (87–89). Only, there is a need for ready-to-use individual testing kits with reasonable prices that can be adapted to clinical laboratories from clinical trials. As mentioned by other authors, lack of “Conformite Europeenne” (CE) or “*In Vitro* Diagnostics” (IVD) labels for the reagents to be used in detection ICs and their counterparts requires more standardization and acceptance of the methods used for the standardization by clinical laboratories that are normally required to use the CE or IVD labeled products only (90). Extensive non-CE and non-IVD panels suggested by the same authors should be evaluated by more laboratories, as the clones chosen for this type of analysis may be different in laboratories, standardization suggestions are cumbersome and challenging with many variations in the measurement systems, reagents, preparation, and analysis methods.

In some solid tissue cancers, after ICI treatment a pseudo progression (an initial increase in tumor volume) may be observed, and pseudo progression should be distinguished from progressive disease for an accurate clinical decision (91). Detailed immunophenotyping of leukemias in a well-structured and standardized framework along with markers of ICP inhibitors will help to make decision on progression and pseudo progression. Employing a full panel of ICIs is suggested, since one marker disappears or is expressed low after treatment while other markers increase as a compensation mechanism.

A study performed for assessment of baseline serological autoantibody profile in prediction of responders to ICIs included lymphoma cases with other solid tumors and found that PD-1 and PD-L1 IgG2 autoantibodies were highly produced in approximately 20% of lymphoma responders. The authors suggest that using high-throughput protein microarrays for selected autoantibody markers can predict anti-PD-1 therapeutic response and guide the therapy (92). To implement this approach, there is a need for more evidence-based studies to confirm the prediction of response to ICIs in leukemias and other hematological malignancies. In a recently published study, measurement of CD8^+^LAG3^+^ cells in peripheral blood of solid tissue tumors were significantly correlated with response, survival and progression-free survival (73). Authors suggested that pretreatment measurement of these cells could be used for identifying actionable immune targets. Clinical trials using this panel and evaluating outcome of patients could provide more insight into use of LAG3+ cells as a marker for clinical decisions (88, 93).

Immunophenotyping of leukemias in pediatric and adult patients differ from several aspects. ACCELERATE and European Medicines Agency Pediatric Strategy Forum for medicinal product development of ICIs has published a statement paper for use of ICIs in pediatric patients and declared that; “The major challenge for developing ICIs for pediatric cancers is the lack of neoantigens (based on mutations) and corresponding antigen-specific T cells. Progress critically depends on understanding the immune macroenvironment and microenvironment and the ability of the adaptive immune system to recognize pediatric cancers in the absence of high neoantigen burden. Future clinical studies regarding administration of ICIs in children need to build upon strong biological hypotheses that take into account the distinctive immunobiology of childhood cancers in comparison to that of ICI responsive adult cancers” (94). Extensive profiling of patients in well-structured clinical trials will help understand immunobiology better and allow reaching to a consensus for follow-up.

In this article, we have focused on the possibilities and challenges ahead in clinical immunology and hematology laboratories. There are much more to be explored in imaging and other laboratories (clinical chemistry, clinical microbiology) along with the genetic laboratories in order to make the most accurate assessments for immunotherapies with ICIs. What we need to do in clinical laboratories is to develop standardized methods for all of the above-mentioned testing, work to offer cheaper and more accessible services to patients and develop new methods to be delivered in routine laboratories. We have come a long way since Rituximab was commercialized more than twenty years ago, and we have learned much about immunity during the COVID-19 pandemic period, but we still need to synchronize the tasks of clinical laboratories with the ever-expanding range of immunotherapies.

## Author Contributions

Drafting manuscript: BA, MS, CP, EG, MG-H, and GYD. Preparing the figures: CP and EG. Editing and revising the manuscript: GYD. All authors contributed to the article and approved the submitted version.

## Conflict of Interest

The authors declare that the research was conducted in the absence of any commercial or financial relationships that could be construed as a potential conflict of interest.

## Publisher’s Note

All claims expressed in this article are solely those of the authors and do not necessarily represent those of their affiliated organizations, or those of the publisher, the editors and the reviewers. Any product that may be evaluated in this article, or claim that may be made by its manufacturer, is not guaranteed or endorsed by the publisher.
