# Supplementary material for: Comparison of Laboratory Methods for the Clinical Follow Up of Checkpoint Blockade Therapies in Leukemia: Current Status and Challenges Ahead
Source: Front Oncol. 2022 Jan 27;12:789728. doi: 10.3389/fonc.2022.789728 (PMC8829140; doi:10.3389/fonc.2022.789728)
Supplement: Supplementary file 1 [file Table_1.docx]

Supplementary Material

**Table S1.** Selected clinical trials involving immune checkpoint inhibitors in the treatment of hematological malignancies including leukemia.

| **Clinical trial number** | **Status** | **Phase** | **Intervention** | **Target** | **Patient Population** |
| --- | --- | --- | --- | --- | --- |
| **-** | Completed | I | CT-011 (1) | PD-1 | Advanced hematological malignancies  *n*: 17, AML:8 |
| NCT01822509 | Completed | I | Ipilimumab (2) | CTLA-4 | Hematologic malignancies with relapse after allo-HCT  *n***:** 28, AML:12 |
| NCT02464657 | Completed | II | Nivolumab + cytarabine/- idarubicin (3) | PD-1 + chemotherapy | AML/high-risk MDS upfront therapy  *n*: 32, AML:30 |
| NCT02708641 | Completed | II | Pembrolizumab (4) | PD-1 | AML post remission > 60 years who are not HCT candidates  *n*: 12 |
| NCT02464657 | Completed | I/II | Nivolumab +idarubicin/cytarabine (7 + 3) induction chemotherapy (3) | PD-1 + chemotherapy | AML and high-risk MDS  *n*: 44, AML:42 |
| NCT02996474 | Completed | I/II | Pembrolizumab + decitabine (5) | PD-1 + chemotherapy | RR-AML  *n*: 10 |
| NCT01822509 | Completed | I/II | Nivolumab + ipilimumab (2) | PD-1 + CTLA-4 | Hematologic malignancies including AML with relapse after HCT  *n*: 28, AML:12 |
| NCT03358719 | Completed | I | Nivolumab + NY-ESO-1 vaccination (Anti-DEC-205-NY-ESO-1 fusion protein + poly-ICLC) + decitabine (6) | PD-1 + tumor vaccine + HMA | Higher-risk MDS, AML with ≤30% blasts  *n*: 8 |
| NCT02663518 | Completed | I | Nivolumab + TTI-621 (anti-CD47 antibody) (7) | PD-1 + CD47 | RR solid or hematologic malignancies including AML  *n*: 164, AML:20 |
| NCT02981914 | Ongoing | I | Pembrolizumab (8) | PD-1 | Hematologic malignancies (including AML) relapsed after allo-HCT |
| NCT03286114 | Ongoing | I | Pembrolizumab | PD-1 | AML/MDS/ALL relapsed after allo-HCT |
| NCT02275533 | Ongoing | II | Nivolumab  Arm I: Nivolumab  Arm II: standard of care | PD-1 | AML in CR |
| NCT02532231 | Ongoing | II | Nivolumab | PD-1 | AML in CR at high risk of relapse |
| NCT02771197 | Ongoing | II | Pembrolizumab + autologous SCT | PD-1 | AML with high risk of relapse not eligible for allo-HCT |
| NCT02768792 | Ongoing | II | Pembrolizumab + high-dose cytarabine (9) | PD-1 +  chemotherapy | RR-AML |
| NCT02890329 | Ongoing | I | Ipilimumab + decitabine | CTLA-4 + HMA | RR MDS/AML |
| NCT02845297 | Ongoing | II | Pembrolizumab + azacytidine (10) | PD-1 + HMA | RR MDS/AML, AML> 65 years |
| NCT02397720 | Ongoing | II | Arm I: Nivolumab + azacytidine (11)  Arm II: Nivolumab + azacitidine + ipilimumab (12) | PD-1 + HMA +/-  CTLA-4 | RR-AML, AML > 65 years |
| NCT02775903 | Ongoing | II | Durvalumab + azactidine (13) | PD-L1 + HMA | High-risk MDS, AML > 65 years |
| NCT02846376 | Ongoing | I | Arm I: Nivolumab  Arm II: Ipilimumab  Arm III: Nivolumab + ipilimumab | PD-1  CTLA-4  PD-1 + CTLA-4 | AML after allo-HCT |
| NCT03066648 | Ongoing | I | anti-TIM-3 antibody MBG453  anti-PD-1 antibody PDR001  decitabine  Arm I: PDR001 + decitabine  Arm II: MBG453 + decitabine  Arm III: PDR001 +MBG453 + decitabine  Arm IV: MBG453  Arm V: PDR001 +MBG453 (14) | TIM3 + PD-1 + HMA | RR-AML/MDS, AML not eligible for chemotherapy |
| NCT01096602 | Ongoing | II | Pidilizumab + dendritic cell vaccine | PD-1 + Tumor vaccine | AML in CR |
| NCT03248479 | Ongoing | I | Magrolimab + azacytidine (15) | CD47 + HMA | N=43 (18 HR-MDS, 25 AML); all frontline and chemotherapy ineligible |
| NCT03600155 | Ongoing | I | Nivolumab + ipilimumab following allo-HCT | PD-1 + CTLA-4 | HR-AML or MDS with relapsed or refractory disease following allo-HCT |
| NCT02530463 | Ongoing | II | Nivolumab +/- ipilimumab +/- azacitidine | PD-1 +/- CTLA-4 +/- HMA | Untreated and HMA-failure MDS |

*Allo: allogeneic, AML: acute myeloid leukemia, CR: complete remission, HCT: hematopoietic stem cell transplantation, HMA: hypomethylating agent, HR: high risk, MDS: myelodisplasic syndrome, RR: relapsed/refractory.*

**References**

1. Berger R, Rotem-Yehudar R, Slama G, Landes S, Kneller A, Leiba M, et al. Phase I safety and pharmacokinetic study of CT-011, a humanized antibody interacting with PD-1, in patients with advanced hematologic malignancies. **Clin Cancer Res** (2008) 14(10):3044-51.

2. Davids MS, Kim HT, Bachireddy P, Costello C, Liguori R, Savell A, et al. Ipilimumab for Patients with Relapse after Allogeneic Transplantation. **N Engl J Med** (2016) 375(2):143-53.

3. Ravandi F, Assi R, Daver N, Benton CB, Kadia T, Thompson PA, et al. Idarubicin, cytarabine, and nivolumab in patients with newly diagnosed acute myeloid leukaemia or high-risk myelodysplastic syndrome: a single-arm, phase 2 study. **Lancet Haematol** (2019) 6(9):e480-e8.

4. Winer ES, Stone RM. Novel therapy in Acute myeloid leukemia (AML): moving toward targeted approaches. **Ther Adv Hematol** (2019) 10:2040620719860645.

5. Lindblad KE, Thompson J, Gui G, Valdez J, Worthy T, Tekleab H, et al. Pembrolizumab and Decitabine for Refractory or Relapsed Acute Myeloid Leukemia. **Blood** (2018) 132(Supplement 1):1437-.

6. Lamble AJ, Lind EF. Targeting the Immune Microenvironment in Acute Myeloid Leukemia: A Focus on T Cell Immunity. **Front Oncol** (2018) 8:213.

7. Ansell SM, Maris MB, Lesokhin AM, Chen RW, Flinn IW, Sawas A, et al. Phase I Study of the CD47 Blocker TTI-621 in Patients with Relapsed or Refractory Hematologic Malignancies. **Clin Cancer Res** (2021) 27(8):2190-9.

8. Kline J, Liu H, Michael T, Artz AS, Godfrey J, Curran EK, et al. Pembrolizumab for the Treatment of Disease Relapse Following Allogeneic Hematopoietic Cell Transplantation. **Blood** (2018) 132(Supplement 1):3415-.

9. Zeidner JF, Vincent BG, Esparza S, Ivanova A, Moore DT, Foster MC, et al. Final Clinical Results of a Phase II Study of High Dose Cytarabine Followed By Pembrolizumab in Relapsed/Refractory AML. **Blood** (2019) 134(Supplement_1):831-.

10. Gojo I, Stuart RK, Webster J, Blackford A, Varela JC, Morrow J, et al. Multi-Center Phase 2 Study of Pembroluzimab (Pembro) and Azacitidine (AZA) in Patients with Relapsed/Refractory Acute Myeloid Leukemia (AML) and in Newly Diagnosed (≥65 Years) AML Patients. **Blood** (2019) 134(Supplement_1):832-.

11. Daver N, Garcia-Manero G, Basu S, Boddu PC, Alfayez M, Cortes JE, et al. Efficacy, Safety, and Biomarkers of Response to Azacitidine and Nivolumab in Relapsed/Refractory Acute Myeloid Leukemia: A Nonrandomized, Open-Label, Phase II Study. **Cancer Discov** (2019) 9(3):370-83.

12. Garcia-Manero G, Sasaki K, Montalban-Bravo G, Daver NG, Jabbour EJ, Alvarado Y, et al. A Phase II Study of Nivolumab or Ipilimumab with or without Azacitidine for Patients with Myelodysplastic Syndrome (MDS). **Blood** (2018) 132(Supplement 1):465-.

13. Zeidan AM, Cavenagh J, Voso MT, Taussig D, Tormo M, Boss I, et al. Efficacy and Safety of Azacitidine (AZA) in Combination with the Anti-PD-L1 Durvalumab (durva) for the Front-Line Treatment of Older Patients (pts) with Acute Myeloid Leukemia (AML) Who Are Unfit for Intensive Chemotherapy (IC) and Pts with Higher-Risk Myelodysplastic Syndromes (HR-MDS): Results from a Large, International, Randomized Phase 2 Study. **Blood** (2019) 134(Supplement_1):829-.

14. Borate U, Esteve J, Porkka K, Knapper S, Vey N, Scholl S, et al. Phase Ib Study of the Anti-TIM-3 Antibody MBG453 in Combination with Decitabine in Patients with High-Risk Myelodysplastic Syndrome (MDS) and Acute Myeloid Leukemia (AML). **Blood** (2019) 134(Supplement_1):570-.

15. Sallman DA, Al Malki M, Asch AS, Lee DJ, Kambhampati S, Donnellan WB, et al. Tolerability and efficacy of the first-in-class anti-CD47 antibody magrolimab combined with azacitidine in MDS and AML patients: Phase Ib results. **Journal of Clinical Oncology** (2020) 38(15_suppl):7507-.
